# Inhibition of mitochondrial respiration by general anesthetic drugs

**DOI:** 10.1007/s00210-022-02338-9

**Published:** 2022-11-17

**Authors:** Anton Fedorov, Alina Lehto, Jochen Klein

**Affiliations:** Department of Pharmacology and Clinical Pharmacy, College of Pharmacy, Goethe University Frankfurt, Max-Von-Laue-Str. 9, 60438 Frankfurt, Germany

**Keywords:** Isoflurane, Sevoflurane, Halothane, Ketamine, Propofol, Pentobarbital

## Abstract

General anesthetic drugs have been associated with various unwanted effects including an interference with mitochondrial function. We had previously observed increases of lactate formation in the mouse brain during anesthesia with volatile anesthetic agents. In the present work, we used mitochondria that were freshly isolated from mouse brain to test mitochondrial respiration and ATP synthesis in the presence of six common anesthetic drugs. The volatile anesthetics isoflurane, halothane, and (to a lesser extent) sevoflurane caused an inhibition of complex I of the electron transport chain in a dose-dependent manner. Significant effects were seen at concentrations that are reached under clinical conditions (< 0.5 mM). Pentobarbital and propofol also inhibited complex I but at concentrations that were two-fold higher than clinical EC_50_ values. Only propofol caused an inhibition of complex II. Complex IV respiration was not affected by either agent. Ketamine did not affect mitochondrial respiration. Similarly, all anesthetic agents except ketamine suppressed ATP production at high concentrations. Only halothane increased cytochrome c release indicating damage of the mitochondrial membrane. In summary, volatile general anesthetic agents as well as pentobarbital and propofol dose-dependently inhibit mitochondrial respiration. This action may contribute to depressive actions of the drugs in the brain.

## Introduction

General anesthetic drugs (GAD) are of utmost importance in medical practice as they enable surgical procedures in the absence of pain and consciousness. Anesthesia is a complex situation in which increases of GABA_A_ receptor activity cause central depression whereas inhibition of NMDA receptors is associated with analgesia and amnesia (Rudolph and Antkowiak 2004; Franks 2008). Both mechanisms contribute to post-surgical cognitive dysfunction that is often observed in elderly patients. All GAD also have certain unwanted effects including cardiac depression and reductions of blood pressure. Early work has also identified an interference with mitochondrial respiration and the handling of calcium by GAD (Cohen 1973), and more recent work links mitochondrial actions to the therapeutic actions of volatile anesthetic drugs (Zimin et al. 2016, 2018; Jung et al. 2022). Pro-apoptotic effects of GAD were described in animals of very young age which were accompanied by impaired neurogenesis, neuronal cell death, and long-term cognitive dysfunctions (Jevtovic-Todorovic et al. 2003; Creeley and Olney 2010); and a vivid discussion has ensued if these observations should have impact on clinical practice (Istaphanous and Loepke 2009; Vutskits and Xie 2016). In addition, mitochondrial toxicity was also discussed in the context of patients with mitochondrial defects (Niezgoda and Morgan, 2013) and in aged patients (Iqbal et al. 2019; Belrose and Noppens 2019).

We have previously observed that lactate levels increase when mice sustained general anesthesia (Horn and Klein 2010; Schwarzkopf et al. 2013). Using micro-dialysis in live mice, we measured a four-fold increase of lactate levels during anesthesia with volatile anesthetics such as halothane, isoflurane, and sevoflurane. In contrast, lactate levels remained unchanged when mice were anesthetized with injectable anesthetics such as propofol, pentobarbital,or ketamine (Horn and Klein 2010). As the well-known inhibition of mitochondrial function would explain these increases of lactate, we prepared mitochondria from healthy mouse brain and compared the effects of six GAD on the activities of the mitochondrial electron transport chain and on ATP synthesis. Our results confirm that volatile anesthetic drugs inhibit mitochondrial function in vitro, a result that corroborates our previous in *vivo*-findings. Mitochondria were particularly sensitive to volatile anesthetics and less or not affected by injectable anesthetics.

## Materials and methods

### Animals

Female CD-1 mice (29-32 g, Charles River) were used for the experiments. They were kept in standard cages, under 60% humidity, 22° C temperature, and a 12-h light/dark cycle. Food and water were available ad lib. In accordance with GV-Solas guidelines, all procedures were designed to minimize the suffering of the experimental animals. In total, 90 mice were used for this study.

### Isolation of mitochondria

After decapitation, the brain was immediately dissected from the skull, the cerebellum was removed, and the remaining brain tissue (ca. 300 mg) was homogenized in 2-mL MiR05 buffer (Schwarzkopf et al. 2015; Gnaiger 2020). In addition, a protease inhibitor cocktail was added to the medium (cOmplete Tablets EASY pack, Roche, Mannheim, Germany). The homogenate was centrifuged twice to remove all cell debris (1.400 g, 7 min, 4 °C). The purified supernatant was then centrifuged again (10.000 g, 5 min, 4 °C), and the resulting pellet containing the mitochondria was resuspended in 1-mL MiR05 + PI and centrifuged once again (1.400 × g, 3 min, 4 °C). Finally, the supernatant was centrifuged one more time (10.000 g, 5 min, 4 °C) and the pellet resuspended in 250-µL MiR05 + PI.

### Measurement of mitochondrial respiration

**Fig. 1:**
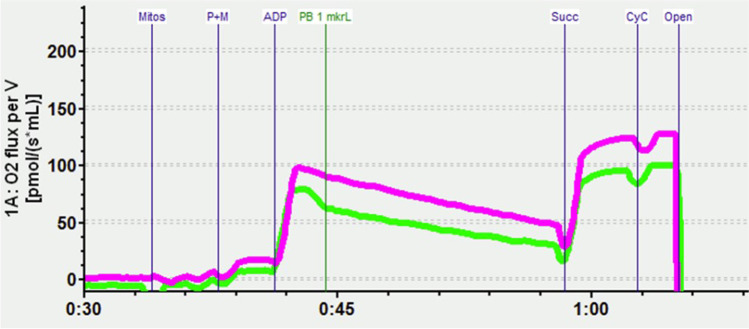
Exemplary readout of the respirometer: oxygen consumption in isolated brain mitochondria, here induced by addition of pyruvate and malate (P + M), ADP, addition of pentobarbital (PB), succinate (Succ), and addition of cytochrome c (CyC) to check for CyC loss induced by pentobarbital. Mauve line, control; green line, pentobarbital

Mitochondria from two hemispheres of the same mouse brain were put into parallel chambers of the respirometer (Oroboros® Instruments, Innsbruck, Austria). Each chamber was filled with 2.4-mL MiR05 medium according to manufacturer’s instructions and kept at 37 °C with constant stirring (750 rpm). After 30 min equilibration and subsequent air calibration, 80 µL of the mitochondrial suspensions was injected into the closed chamber. The remaining mitochondria were frozen in liquid nitrogen for protein determination with the Bradford assay. After equilibration, a solution containing pyruvate (5 mM) and malate (1 mM), two substrates linked to complex I (CI), was injected into the chamber (LEAK-state, non-phosphorylating resting state). Then, ADP (2 mM) was added to stimulate oxidative phosphorylation (OXPHOS-CI; ADP-stimulated and CI-linked respiration). To induce the full ADP-stimulated respiration, succinate (10 mM), a CII-linked substrate, was injected (total OXPHOS capacity). To verify the integrity of the outer mitochondrial membrane, cytochrome c (10 µM) was added; mitochondria whose respiration increased by more than 15% upon cytochrome c addition were discarded. The maximum capacity of the electron transfer system (ETS) was determined by the stepwise titration of the uncoupler FCCP (state E). To measure CII respiration, the complex I inhibitor rotenone (2.5 µM) was added (CII-linked substrate state, uncoupled). After inhibition of complex III by antimycin A (2.5 µM), the residual oxygen consumption (ROX; ROX-state) remains, which is used to correct the mitochondrial respiration states. Ascorbate (2 mM) and tetramethyl-phenylendiamine (TMPD, 0.5 mM) are artificial electron donors that induce maximum cytochrome c- oxidase (complex IV, CIV) respiration by reducing cytochrome c. Ascorbate regenerates TMPD and is injected first. At the end of the experimental run, CIV is inhibited by a high concentration of sodium azide (120 mM). The chemical background as well as ROX remains. To obtain the CIV activity, this value has to be subtracted from the total measured oxygen flux (for further details, see Schwarzkopf et al. 2015). A shortened version of the titration protocol is shown in Fig. 1.

Isoflurane, sevoflurane, and halothane are volatile. Therefore, the incubation of the sample suspension was carried out in closed glass vials to prevent evaporation of the drugs during incubation. The concentrations of the volatile anesthetic drugs can be calculated from the minimal alveolar concentration and the blood/gas coefficient and was taken from the literature (see legend of Table 1).

**Table 1:** Comparison of blood concentrations of anesthetic drugs and EC_50_ values and complex I inhibition in the brain

Anesthetic	Blood conc. (EC_50_) (literature data)	Complex I inhibition (present work; only *p* < 0.05)
Isoflurane	0.32 mM^1^	0.20 mM
Sevoflurane	0.35 mM^1^	0.38 mM
Halothane	0.34 mM^1^	0.47 mM
Propofol	45 µM^2^	84 µM
Pentobarbital	53 µM^3^	120 µM
Ketamine	1 µM^4^	n.s

^1^Nickalls and Mapleson 2003; Rudolph & Antkowiak 2004. ^2^Davidson et al. 1993. ^3^Wan et al. 2003. ^4^Peltoniemi et al. 2016

### ATP measurement

Glass vials with MiRO5 medium were kept at 37 °C on an aluminum heating block. Malate (2 mM), pyruvate (5 mM), and ADP (2 mM) were added and vials were incubated for about 2 min. Afterward, isoflurane (1.20 mM), sevoflurane (3.42 mM), halothane (2.26 mM), ketamine (7.8 µM), pentobarbital (360.0 µM), propofol (252.0 µM), and sample suspension (60 µl) were added to the vials. Incubation was carried out for 15 min in a shaking water bath at 37 °C. Then, the reaction was stopped on ice, and ATP content was measured using an ATP Assay Kit (Lonza). For this purpose, ice-cold samples were diluted 1:1000 with MiRO5 buffer. 100 µl of diluted samples was shaken (Unimax 1010) with lysis buffer for 10 min at room temperature in black 96-well plates. Next, the ATP measurement reagent (100 µl) was added and the mixture was incubated for 15 min in a dark place at room temperature. Measurement of fluorescence was carried out with a Wallac Victor2 multi-label counter (PerkinElmer). Exposition time was 0.5 s per well. Each sample was measured in 3 different wells and each well plate was measured three times. Luminescence intensity of treated samples was compared with reference samples.

### Statistical analysis

**Fig. 2:**
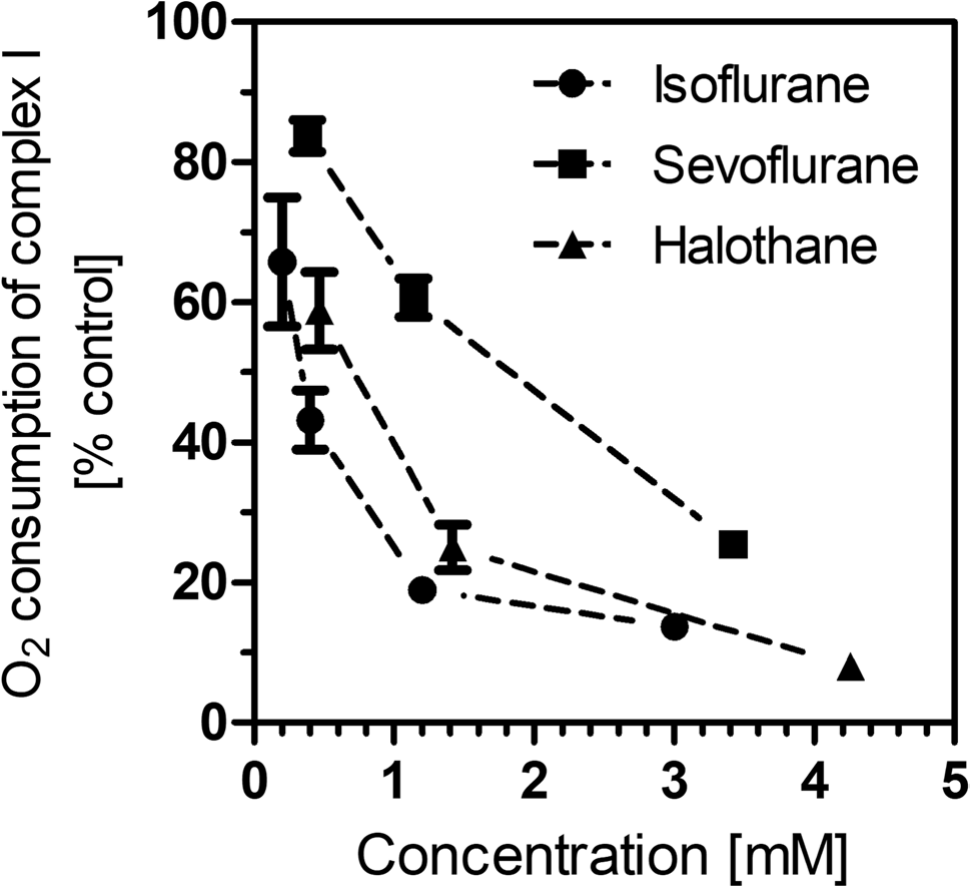
Oxygen consumption by complex I in isolated brain mitochondria in the presence of isoflurane, sevoflurane, and halothane. All data points are means ± S.E.M. of *N* = 4–5 experiments. Statistics: one-way ANOVA for each drug with Dunnett’s post-test vs. controls, all data points are significantly different from control incubations (100 ± 2.3%, *N* = 12)

**Fig. 3:**
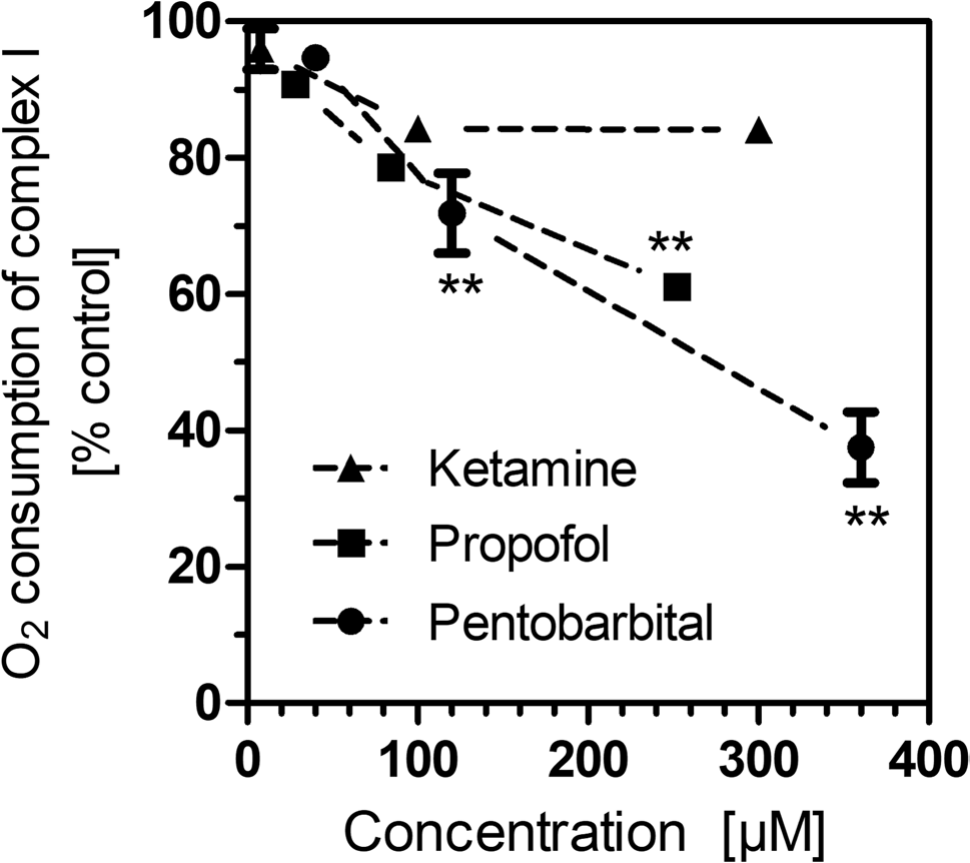
Oxygen consumption by complex I in isolated brain mitochondria in the presence of ketamine, propofol, and pentobarbital. All data points are means ± S.E.M. of *N* = 4 experiments. Statistics: one-way ANOVA for each drug with Dunnett’s post-test vs. controls (100 ± 2.6%, *N* = 12). ^**^, *P* < 0.01 vs. control incubations without drug

**Fig. 4:**
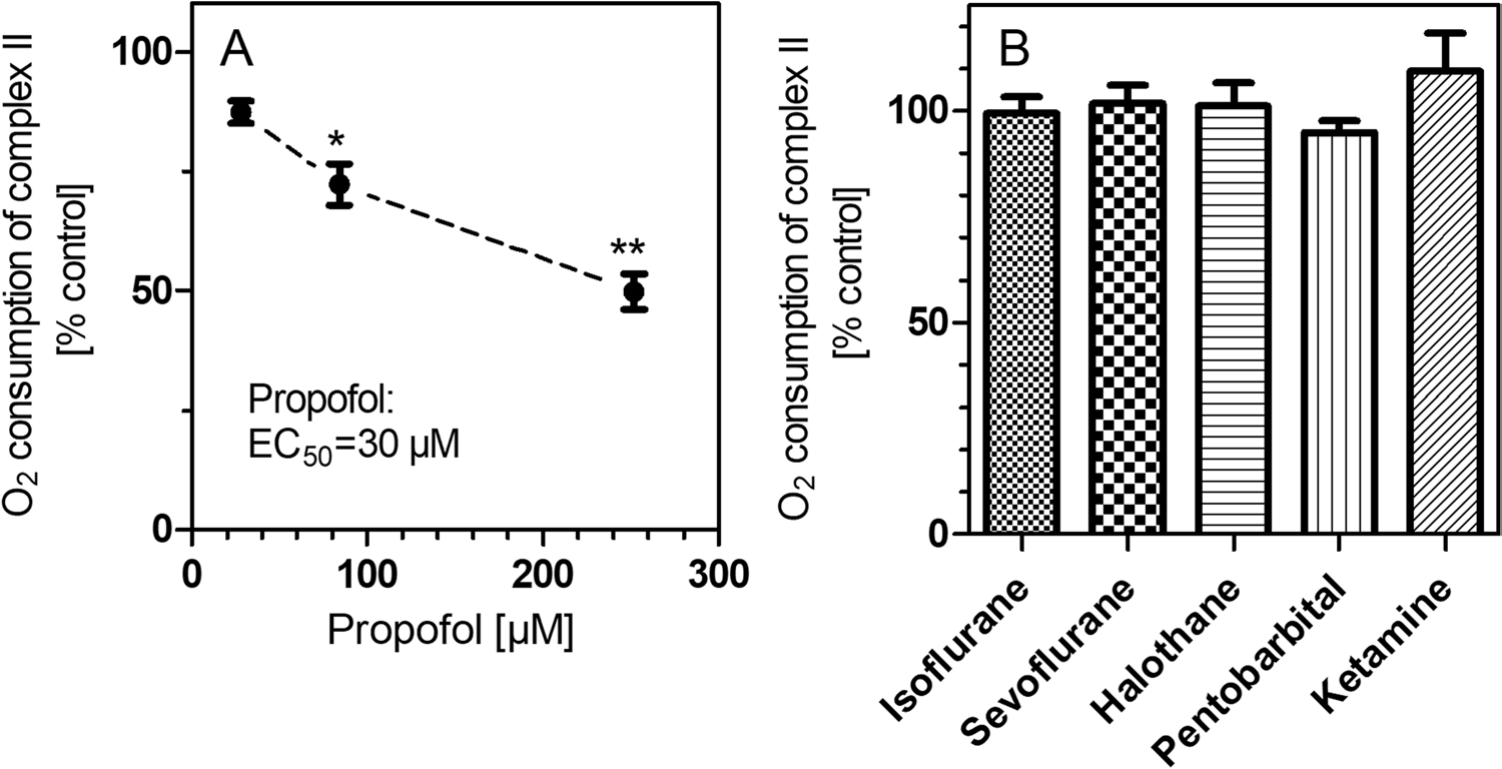
Oxygen consumption by complex II in isolated brain mitochondria in the presence of six anesthetic drugs. (**A**) Inhibition of respiration by propofol. (**B**) Non-significant effects of the remaining five anesthetic drugs. All data points are means ± S.E.M. of *N* = 4 experiments. Statistics: one-way ANOVA with Dunnett’s post-test vs. controls (100 ± 2.1%, *N* = 12). ^*^, *P* < 0.05; ^**^, *P* < 0.01 vs. control incubations without drug

**Fig. 5:**
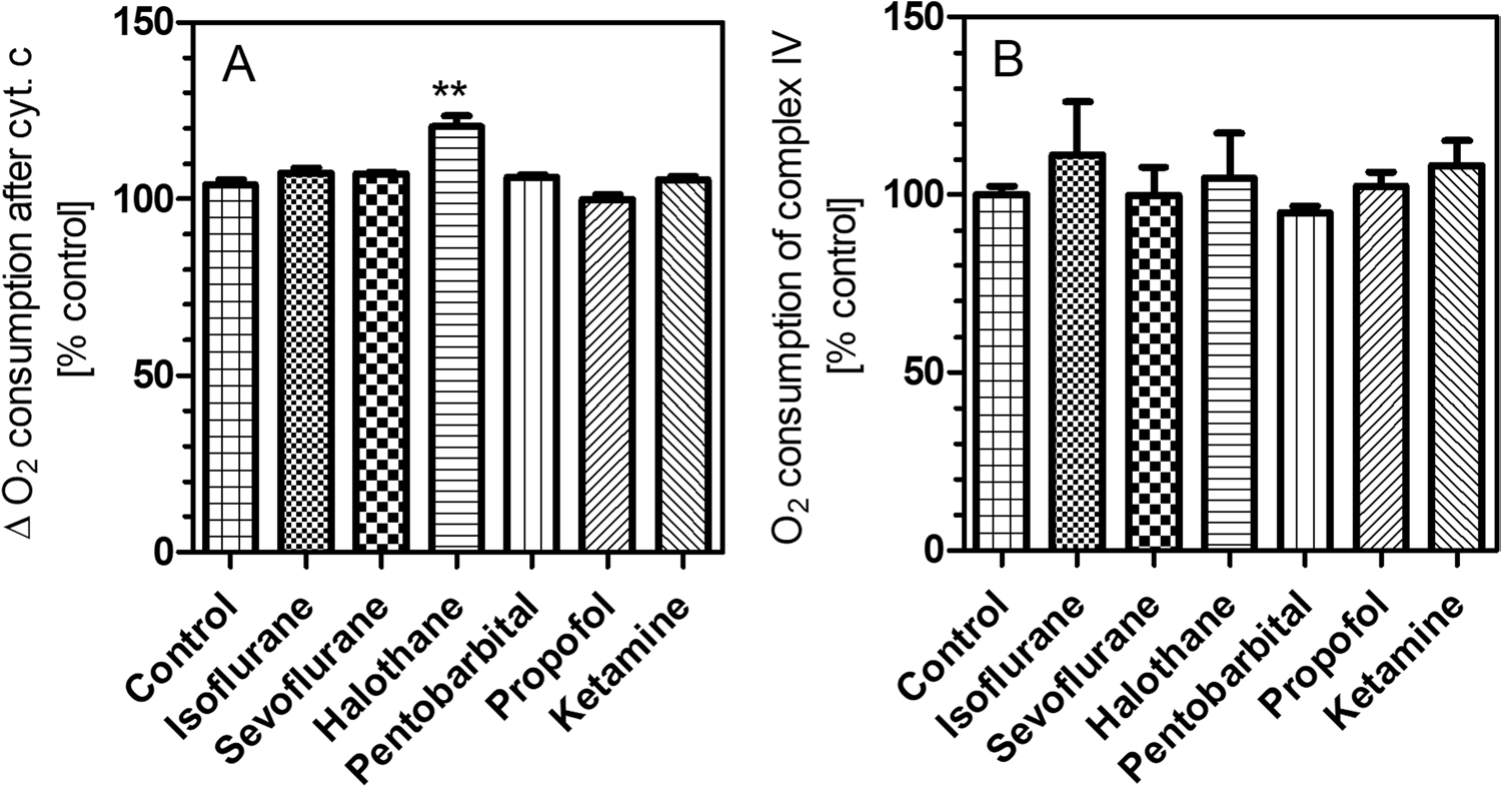
Oxygen consumption in isolated brain mitochondria in the presence of six anesthetic drugs. (**A**) Increase of respiration after addition of cytochrome c. (**B**) Non-significant effects of the six anesthetic drugs on complex IV activity. In this graph, the following concentrations are shown: isoflurane 1.2 mM, sevoflurane 3.42 mM, halothane 4.26 mM, pentobarbital 360 µM, ketamine 7.8 µM, and propofol 254 µM. All data points are means ± S.E.M. of *N* = 4 experiments. Statistics: one-way ANOVA with Dunnett’s post-test vs. controls (100 ± 1.2%, *N* = 12). ^*^, *P* < 0.05; ^**^, *P* < 0.01 vs. control incubations without drug

**Fig. 6:**
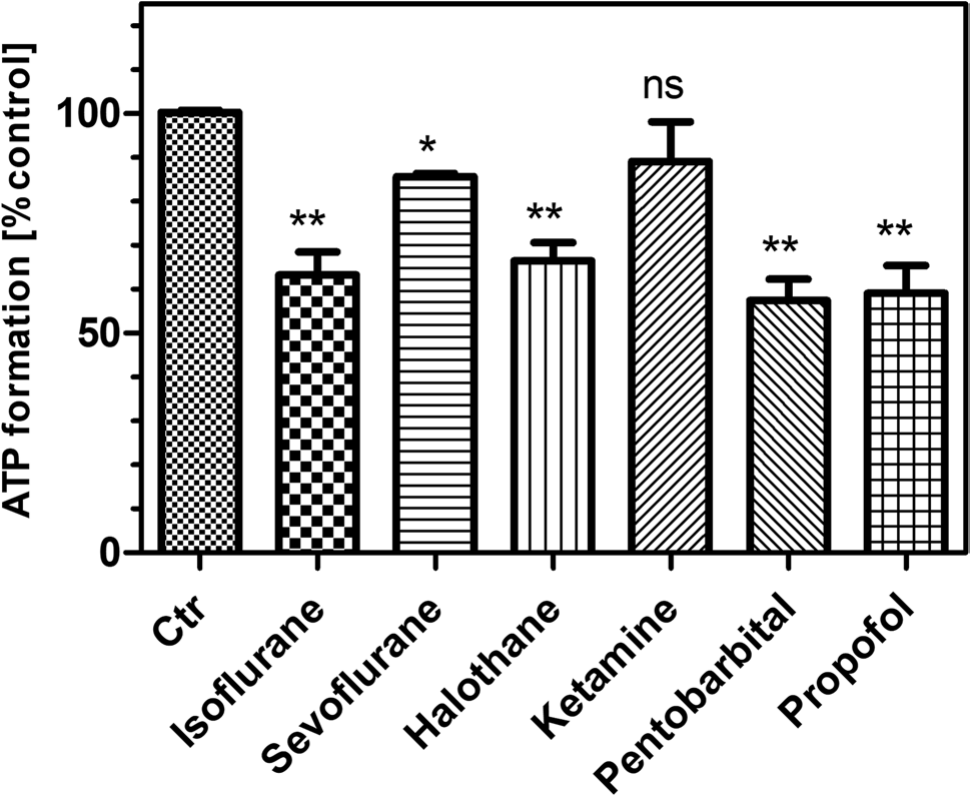
Formation of ATP in isolated brain mitochondria in the presence of six anesthetic drugs. The ATP assay was performed as described in Methods. In this graph, the following concentrations are shown: isoflurane 1.2 mM, sevoflurane 3.42 mM, halothane 4.26 mM, pentobarbital 360 µM, ketamine 7.8 µM, and propofol 254 µM. All data points are means ± S.E.M. of *N* = 4 experiments. Statistics: one-way ANOVA with Dunnett’s post-test vs. controls (Ctr: 100 ± 1.2%, *N* = 12). ^*^, *p* < 0.05; ^**^, *p* < 0.01 vs. control incubations without drug

Data in Figs. 2, 3, 4, 5 and 6 were calculated as percentages of baseline levels (100%). All data are given as means ± S.E.M. of *N* experiments and were analyzed by one-way ANOVA (GraphPad^R^ Prism 5.03) with Bonferroni’s post-test. One-way ANOVA followed by Dunnett’s test was used for the ATP data (Fig. 6). *P* values < 0.05 were considered to be statistically significant.

## Results

In isolated mitochondria from mouse brain, oxygen consumption was measured after addition of suitable substrates (pyruvate and malate for complex I, ADP for oxidative phosphorylation, succinate for complex II, and TMPD for complex IV) and inhibitors (rotenone for complex I, antimycin A for complex III, and azide for complex IV) as described in Methods. In the presence of volatile anesthetics, oxygen consumption by complex I was significantly less than in control incubations (Fig. 2). This effect was particularly strong at high concentrations but was already present in the lowest concentrations which were close to the concentrations used in clinical anesthesia (< 0.5 mM). Sevoflurane was less active than isoflurane and halothane.

When injectable anesthetics were tested, pentobarbital and propofol also strongly reduced complex I respiration (Fig. 3). In contrast, ketamine did not significantly affect complex I respiration.

The calculations of complex II activities showed that only propofol caused a significant inhibition of succinate oxidation (Fig. 4A). The other anesthetic drugs were not active on complex II (Fig. 4B). In this graph, the following concentrations are shown: isoflurane 1.2 mM, sevoflurane 3.42 mM, halothane 4.26 mM, pentobarbital 360 µM, and ketamine 7.8 µM.

When the mitochondrial membrane is damaged or mitochondria depolarize, cytochrome c is released and initiates apoptotic cell death. By adding exogenous cytochrome c, the quality of the mitochondrial preparation can be checked: the increase of oxygen consumption after addition of cytochrome c should be less than 10% (Gnaiger 2020). In the present experiments, only halothane-treated mitochondria showed a higher response to cytochrome c (+ 20.6 ± 3.0%), indicating mitochondrial membrane damage by the drug (Fig. 5A).

Cytochrome oxidase (complex IV) activity was measured after addition of TMPD and ascorbic acid. As shown in Fig. 5B, none of the anesthetic drugs affected complex IV activity.

In separate incubations, we determined the formation of ATP in isolated brain mitochondria in the absence and presence of anesthetic drugs. As shown in Fig. 6, five out of six anesthetics reduced ATP formation. The pattern of effect followed exactly the inhibition of complex I activity as sevoflurane was less active than isoflurane or halothane, and ketamine did not affect ATP formation at all. The effect of propofol was equally strong as that of pentobarbital, probably because inhibitions of complex I and II were additive for this drug.

## Discussion

Inhibition of mitochondrial function has long been acknowledged as a toxic effect of general anesthetics, even before mitochondrial function was fully understood (Cohen 1973). More recent work shows that inhibition of mitochondrial respiration also contributes to the anesthetic action of these compounds (Zimin et al. 2016, 2018; Jung et al. 2022). Mitochondrial effects were reported in early studies for older anesthetic drugs such as diethyl ether, barbiturates, and halothane (Muravchik and Levy 2006). Newer work also concerned isoflurane and sevoflurane that were found to inhibit NADH oxidation in cardiac myocytes (Hanley et al. 2002) and propofol that reduced respiration in synaptosomes (Marian et al. 1997; Bains et al. 2009). In the clinic, this impairment of respiration may contribute to anesthetic effects or to confusional states that are often observed post-surgically in elderly patients (Belrose and Noppens 2019). It may also contribute to neuronal apoptosis in immature brains (see Introduction) or to pre-conditioning effects that were observed for volatile anesthetics, e.g., in the heart (Ye et al. 2012). As previous work often focused on the effect of individual (volatile or injectable) anesthetic drugs, we decided to compare six common anesthetic agents in the same assay procedure. We used respirometry in isolated mitochondria from mouse brain to determine their effects on mitochondrial respiration and ATP synthesis. Anesthetics such as sevoflurane and propofol are in routine clinical use whereas some of the others (e.g., pentobarbital and halothane or isoflurane) are often used in animal studies. The selection of anesthetics followed our earlier study on brain lactate levels (Horn and Klein 2010). The results partly support and extend previous observations made in mitochondrial preparations from different organs and species and using different methodologies to measure mitochondrial respiration.

While halothane, isoflurane, and sevoflurane are all volatile anesthetics, isoflurane is the best-characterized GAD with respect to mitochondrial function. It was shown to inhibit complex I in mitochondria from rat heart (Pravdic et al. 2012) and in hippocampal slices (Zimin et al. 2018). In our previous work, we found that isoflurane caused a rapid, four-fold increase of lactate in mouse brain measured by micro-dialysis (Horn and Klein 2010). This effect was accompanied by increases of glucose and lactate in blood whereas brain glucose levels remained unchanged (Schwarzkopf et al. 2013). Halothane had similar effects as isoflurane whereas sevoflurane had less effects on glucose and lactate. In our present work, both halothane and isoflurane were strong inhibitors of complex I of the mitochondrial electron transport chain and of ATP synthesis whereas sevoflurane caused a significant but weaker inhibition. Halothane was also found to increase cytochrome c release from mitochondria, a toxic action that is likely caused by a reduction of the mitochondrial membrane potential. Our results show that the range of mitochondrial toxicity is halothane > isoflurane > sevoflurane and support the elimination of halothane from clinical practice and the preference for sevoflurane over isoflurane. This conclusion is supported by previous findings in cardiac mitochondria (Hanley et al. 2002) and in synaptosomes (Bains et al. 2006).

In our previous work, injectable GAD such as pentobarbital and propofol caused stronger respiratory depression than volatile GAD but no increase of lactate in brain or blood (Horn and Klein 2010; Schwarzkopf et al. 2013). Nevertheless, we here found impairments of mitochondrial function for both drugs accompanied by reduced ATP synthesis, at least at higher concentrations. Propofol in particular affected complex I as well as complex II of the ETC; it was the only GAD to reduce respiration at complex II. This is in agreement with previous studies in swine cortex in which propofol, but not isoflurane, inhibited substrate flux through the Krebs cycle (Kajimoto et al. 2014) and in hippocampal slices in which propofol reduced oxygen consumption (Berndt et al. 2018). In liver mitochondria, however, propofol was reported to stimulate complex II activity (Felix et al. 2017). Mitochondrial toxicity may also underlie the well-known propofol infusion syndrome (Kam and Cardone 2007) but it should be noted that propofol’s adverse effects in the present study are strongly dose-dependent and occur mostly with high dosages and long exposition times.

Finally, ketamine is a frequently used drug that did not change lactate levels in our previous work (Horn and Klein 2010; the increase in glucose was due to xylazine). One previous publication reported an interference of ketamine with complex I but this work was challenged (Venâncio et al. 2015; Morgan et al. 2016). In our hands, ketamine was the only anesthetic drug that did not significantly affect mitochondrial respiration or ATP synthesis. A direct effect of ketamine on mitochondrial respiration can therefore be excluded.

What is the clinical significance of the present study? It is difficult to determine the concentrations of volatile anesthetics in the brain and on the cellular level because data to that effect do not exist. For the present study, we used blood plasma concentrations of the drugs that can be found in the literature (calculated from clinically determined minimal alveolar concentrations and blood/gas coefficients). Comparison of these concentrations with those that caused a significant inhibition of mitochondrial respiration (Table 1) shows that mitochondrial effects can be expected in the therapeutic range of volatile anesthetics. The same analysis for pentobarbital and propofol suggests that these anesthetic drugs only inhibit mitochondrial respiration at relatively high concentrations, e.g., two times the EC_50_ values. Ketamine, the most potent of the tested drugs, is inactive.

In summary, we used respirometry to compare the effects of six different anesthetic drugs on mitochondria isolated from mouse brain. Isoflurane and halothane as well as pentobarbital strongly impaired complex I activity and reduced ATP synthesis. Halothane was the only compound that also increased cytochrome c release, a toxic effect. Propofol had similar effects but showed inhibition in both complexes I and II. Sevoflurane was less active than isoflurane or halothane. Ketamine did not interfere with mitochondrial respiration. Our results contribute to the understanding of specific anesthetic agents and their mitochondrial effects in human populations.

## Data Availability

The original data can be obtained from the corresponding author by reasonable request.
